# *Polygonum cuspidatum* stem extract (PSE) ameliorates dry eye disease by inhibiting inflammation and apoptosis

**DOI:** 10.20463/jenb.2019.0026

**Published:** 2019-12-31

**Authors:** Bongkyun Park, Kyuhyung Jo, Tae Gu Lee, Ik Soo Lee, Jin Sook Kim, Chan-Sik Kim

**Affiliations:** 1 Clinical Medicine Division, Korea Institute of Oriental Medicine, Daejeon Republic of Korea; 2 Herbal Medicine Research Division, Korea Institute of Oriental Medicine, Daejeon Republic of Korea; 3 Korean Convergence Medicine, University of Science Technology, Daejeon Republic of Korea

**Keywords:** *Polygonum cuspidatum*, dry eye disease, inflammation, apoptosis, human conjunctival cell

## Abstract

**[Purpose]:**

Here, we aimed to determine the effect of *Polygonum cuspidatum* stem extract (PSE) on exorbital lacrimal gland-excised rat models and hyperosmotic stress-stimulated human conjunctival cells (HCCs).

**[Methods]:**

Seven week old male Wistar rats were divided into six groups. Only the rats in the control group (NOR, n=5) did not undergo surgery. Three days after the surgery, the exorbital lacrimal gland-excised rats were randomly allocated to five groups: (1) vehicle-treated dry-eyed rats (DED, n=5); (2) PSE (10 mg/kg) treated DED rats (PSE-10, n=5); (3) PSE (100 mg/kg) treated DED rats (PSE-100, n=5); and (4) PSE (250 mg/kg) treated DED rats (PSE-250, n=5). In addition, the HCC line was co-treated with hyperosmolar media (528 mOsm) and PSE (1-100 μg/ml).

**[Results]:**

PSE treatment restored the tear volume and goblet cell density by inhibiting severe corneal irregularities and damage. The treatment with PSE significantly attenuated the hyperosmolar stress-induced inflammation and cell death through the suppression of mRNA expression levels of Tumor necrosis factor-α (TNF-α), Interleukin-6 (IL-6), Interleukin-1β (IL-1β), and Interferon-γ (IFN-γ), and the expression of Bcl-2-associated X protein (Bax) as well as the activation of caspase-3 *in vitro*.

**[Conclusion]:**

The inhibitory effects of PSE treatment on dry eye disease indicate the potential of nutritional intervention by PES against inflammatory diseases without adverse effects.

## INTRODUCTION

Dry eye disease (DED) leads to damage to the ocular surface simply because of the lack of tears and excessive evaporation of the tear film. However, it has recently been shown to be a multifactorial disease of the ocular surface that is caused by increased osmotic pressure on the tear film, resulting in eye discomfort, which is accompanied by inflammation of the eye surface, visual impairment, and lack of tears causing instability of the tear film^1,2^. The tears can cause dry eyes in two ways: through the lack of tears and because of the abnormal tear layer; often, both of these occur together and lead to dry eyes^3^.

DED is a common disease, from which over 40 million people suffer globally^4^. Domestically, 20%-30% of outpatients who visit ophthalmologists are reported to have DED in epidemiological studies^5^. In addition, the prevalence of DED continues to increase due to several causes, including increased air conditioner use, air pollution, television viewing, increased time on computers, increased use of contact lenses, more refractive surgeries, and an increasing number of elderly people^6,7^. Currently, the treatment for dry eye disease includes the use of artificial eye drops, anti-inflammatory drugs, and surgical methods, but the basic treatment remains incomplete and has disadvantages and various side effects.

A number of food materials or natural compounds extracted from edible plants and have exhibited anti-inflammatory effects have been investigated for use as functional health foods or dietary supplements. The extracts and their metabolites from edible plants have been demonstrated to possess various bio-activities such as anti-mycobacterial, anti-inflammatory, analgesic, anti-proliferative, and cytotoxic effects^8,9^. Moreover, they have been employed in natural medicine and as functional health foods and have shown fewer adverse effects than the artificially synthesized products. Nevertheless, the health-promoting properties of the extracts and bioactive compounds in edible plants and their pharmaceutical potential remain unclear.

*Polygonum cuspidatum* is a herbaceous, perennial plant of the genus *Polygonum*, which is found in Asia and North America^10^. It has been used as a traditional herbal medicine for cough, hepatitis, amenorrhea, leucorrhoea, arthralgia, hyperlipidemia, scalding, and bruises^11^. The active components of *P*. *cuspidatum* are in the leaves, stems, and roots. Many studies have been conducted on the leaves and roots of *P*. *cuspidatum*, but studies on the stem of *P*. *cuspidatum*, which is used as food, are inadequate. Therefore, in this study, we investigated the effects of *P*. *cuspidatum* stem aqueous extract (PSE) in an exorbital lacrimal gland-excised rat model and on hyperosmotic stress-stimulated human conjunctival cells (HCCs). We hypothesized that PSE might have protective effects against dry eye in dry eye-induced *in vivo* and *in vitro* models.

## METHODS

### Preparation of *Polygonum cuspidatum* stem extract

The stem component of *P*. *cuspidatum* was provided by Samil. Co. Ltd (Seoul, Korea). Briefly, *P*. *cuspidatum* (700 g) was extracted in distilled water by incubating at 100°C for 4 h, followed by freeze-drying (yield: 6.57%). The PSE was standardized using the reference compounds, polydatin and rutin (Sigma, MO, USA) by high-performance liquid chromatography (HPLC) according to previously described protocols^12^. Briefly, PSE (10 mg) was dissolved in 50% methanol (10 mL). The solution was filtered through a 0.2 μm filter (Millipore, MA, USA) prior to injection. HPLC analysis was performed with an Agilent 1200 HPLC instrument (Agilent Technologies, CA, USA) equipped with a binary pump, vacuum degasser, auto-sampler, column compartment, and diode array detector.

### Animals and Treatment

Seven week old male Wistar rats were purchased from Orient Bio (Seoul, Korea). The rats were anesthetized with the intraperitoneal injection of ketamine (75 mg/kg) and xylazine (10 mg/kg). After anesthesia, the tear lacrimal glands were removed by surgical operation. Rats in the normal control group (NOR, n= 5) were not subjected to surgical operation. After 3 days of performing surgery, the exorbital lacrimal gland-excised rats were randomly allocated to five groups: (1) vehicle-treated dry eyed rats (DED, n=5); (2) 10 mg/kg PSE-treated DED rats (PSE-10, n=5); (3) 100 mg/kg PSE-treated DED rats (PSE-100, n=5); (4) 250 mg/kg PSE-treated DED rats (PSE-250, n=5). The animal experiments were approved by the Institutional Animal Care and Use Committee (IACUC approval No. 18-072).

### Tear volume measurement

Tear volume was measured at day 7 after surgical operation and treatment of PSE. All experiments were conducted according to previously known protocols^13^. Phenol red-impregnated cotton threads (Zone Quick, USA) were held with fine forceps and placed in the lateral canthus for 1 min. The tear volume was then measured under a microscope and expressed as the length of the color-changed threads that absorbed the tear fluid.

### Analysis of corneal irregularity

The corneal irregularity was investigated in rats from each group. Briefly, reflected lines of ring-shaped light from the fiber-optic ring illuminator of a stereomicroscope (SZ51; Olympus, Japan) were lighted on the corneal surface of anesthetized rats, and the reflected lines of the light were captured with a DP21 digital camera (Olympus, Japan). Scores of corneal irregularity were graded according to the number of distorted quadrants in the reflected white ring as follows: 0, no distortion; 1, distortion in one quadrant; 2, distortion in two quadrants; 3, distortion in three quadrants; 4, distortion in all four quadrants; 5, severe distortion in which no ring could be recognized.

### Ocular surface evaluation

All experimental procedures were performed in both eyes of each subject. The data of the eye with the worst damage were collected for analysis. To measure tear breakup time, sodium fluorescein dye was added to the eye, and the tear film was observed under a slit lamp while the rats were prevented from blinking until tiny dry spots developed. Generally, >10 s is considered to be normal, 5-10 s is marginal, and < 5 s is low. To evaluate corneal epithelial defects, the corneas were stained with 3% Lissamine Green (Sigma-Aldrich, MO, USA). Fluorescein score was analyzed as follows: 0, absent; 1, slightly punctate staining in less than 30 spots; 2, punctate staining in more than 30 spots, but without diffusion; 3, severe diffused staining, but without positive plaque; 4, positive fluorescein plaque. The representative images of each scale were provided previously^14^.

### Histology

To evaluate the density of conjunctival goblet cells, conjunctival sections were stained with periodic acid Schiff (PAS) and analyzed using a commercially available kit (Sigma-Aldrich, MO, USA) according to the manufacturer's instructions. The sections were photographed using a virtual microscope (Olympus, Japan). Goblet cell density in the superior and inferior conjunctiva was measured in three sections of each eye and was indicated as the number of goblet cells per 100 μm.

### Cell culture

The human conjunctival cell line HCC was obtained from the American Type Culture Collection (ATCC, VA, USA). The cells were cultured according to the manufacturer’s instruction, in RPMI medium (Welgene Inc., Korea) supplemented with 100 IU/mL penicillin, 100 mg/mL streptomycin, and 10% heat-inactivated fetal bovine serum (FBS), which was purchased from Gibco Invitrogen, CA, USA, in an incubator at 5% CO_2_ and 37°C. For sub-culturing, the cells were detached using 0.125% trypsin containing 0.01 M EDTA (Gibco Invitrogen).

### Quantitative real-time PCR

Following the manufacturer's protocol, the total RNA was extracted from HCC cells using the Trizol Reagent. Isolated RNA (1 mg/ml) was reverse transcribed using Super-Script II kit for cDNA synthesis. The cDNA was subjected to quantitative real-time (qRT)-PCR using thermocyclers from Applied Biosystems (Franklin Lakes, NJ). The sequences of the primers corresponding to the mouse adipogenic genes analyzed in this study are shown in Table 1.

**Table 1.** Realtime PCR primer sequences.

Genes	Sequence	PCR primer
hIL-6	Sense	5’-AAATTCGGTACATCCTCGAC-3’
	antisense	5’-CAGGAACTGGATCAGGACTT-3’
hTNF-α	Sense	5’-TTCTCCTTCCTGCTTGTG-3’
	antisense	5’-CTGAGTGTGAGTGTCTGG-3’
hIFN-γ	Sense	5’-AGGGAAGCGAAAAAGGAGTCA-3’
	antisense	5’-GGACAACCATTACTGGGATGCT-3’
hIL-1β	Sense	5'-CCACAGACCTTCCAGGAGAATG-3'
	antisense	5'-GTGCAGTTCAGTGATCGTACAGG-3'
hGAPDH	Sense	5'-CCAGCCGAGCCACATCGCTC-3'
	antisense	5'-ATGAGCCCCAGCCTTCTCCAT-3'

### Western blot

HCCs were seeded in 60-mm dishes (5 × 10^5^ cells/mL) and co-treated with PSE (1, 10, or 100 μg/ml) for 24 h. The cells were washed with PBS and lysed with RIPA lysis buffer (Invitrogen, CA, USA). After cell lysis, lysates were clarified by centrifugation at 13,000 rpm for 15 min at 4℃. Protein concentration was determined using the Bio-Rad DC protein assay with bovine serum albumin (BSA) as the standard. The whole cell lysates were resolved by sodium dodecyl sulfate-polyacrylamide gel electrophoresis, and were electrophoretically transferred to the PVDF membranes (Amersham, Arlington Hights, IL) and probed with antibodies against Bax, B-cell lymphoma 2 (Bcl-2), Caspase-3, and β-actin (Cell Signaling Technology, MA, USA). The blots were developed using enhanced chemiluminescence (ECL) kit. In all immunoblotting experiments, the blots were reprobed with an anti-β-actin antibody as a loading control. The band intensities were measured using the ImageJ software (NIH, MD, USA).

### Statistical analysis

Representative data from three independent experiments are presented as means ± standard error of the mean (SEM). Comparisons between control and experimental values were analyzed with one-way analysis of variance. Analyses were performed with Prism 7 from GraphPad Software (CA, USA). Statistical significance was defined as p < 0.05.

## RESULTS

**Figure 1.**
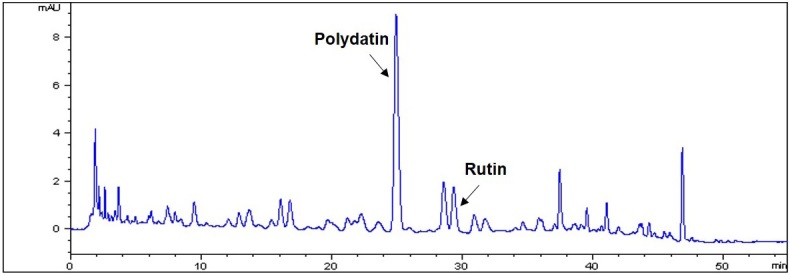
HPLC chromatogram of PSE. Stem aqueous extract of P. cuspidatum at 330 nm.

HPLC analysis was used to investigate the quality of PSE and analyze the active compounds. The UV detector was set at 330 nm for HPLC analysis of two standard compounds: polydatin and rutin. As shown in Figure 1A, we examined the retention times and UV spectral data of PSE as compared to those of the standard compounds. Polydatin and rutin were detected among many peaks of PSE. The results of the comparison between these compounds and PSE showed that PSE also contained two compounds with the same retention times. In addition, the amount of the two compounds in PSE was measured as 1.22 ± 0.02 mg/g and 0.60 ± 0.01 mg/g for polydatin and rutin, respectively (Figure 1B and Table 2).

**Table 2.** The contents of two active components in PSE

Sample name	Content (mg/g)	
	Polydatin	Rutin
PSE	1.22±0.02	0.60±0.01

**Figure 2.**
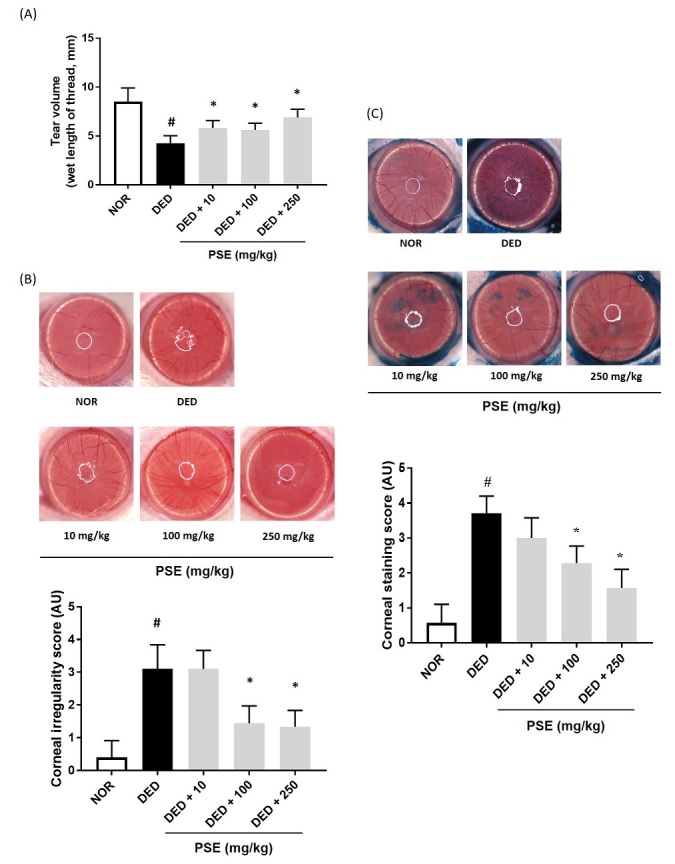
Effects of PSE on dry eye disease *in vivo*. (A) Tear volume was measured using the phenol red thread tear test. (B) Reflected images of a white ring from the fiber-optic ring illuminator of a stereomicroscope. Scale bar is 1 mm; (C) Lissamine green staining and its index. The values in the bar graph represent the mean ± standard error (SE), n = 5. # statistical difference from the normal group (*p*<0.001), *statistical difference from the vehicle-treated dry eyed group (*p*<0.0001).

To determine whether PSE has a protective effect against DED, we used the exorbital lacrimal gland-excised animal model. As shown in Figure 2A, tear fluid volume was remarkably reduced in the excision lacrimal gland (DED, 4.27 ± 0.75, F=27.43, *p*<0.001), as compared to that of the normal group. In contrast, oral PSE administration restored the tear volume in a dose-dependent manner, as compared to that of the DED group (5.83 ± 0.75, 5.63 ± 0.67 and 6.91 ± 0.83, F=27.43, *p*<0.01, respectively). Corneal irregularities and the staining score were examined to investigate the effect of PSE on DED-stimulated corneal tissue damage. DED induced severe corneal irregularities and damage. However, oral administration of PSE at 100 mg/kg and 250 mg/kg to DED group, significantly alleviated the corneal condition, by decreasing the q quantitative score of corneal irregularity (F=41.78, *p*<0.0001) (Figure 2B). Corneal staining using lissamine green showed severe corneal damage in the DED group. In the groups that received oral administration of PSE at 10 mg/kg and 100 mg/kg, the quantitative data were remarkably inhibited to 2.29 ± 0.49 and 1.57 ± 0.53, as compared to those in the DED group (F=37.91, *p*<0.0001) (Figure 2C).

**Figure 3.**
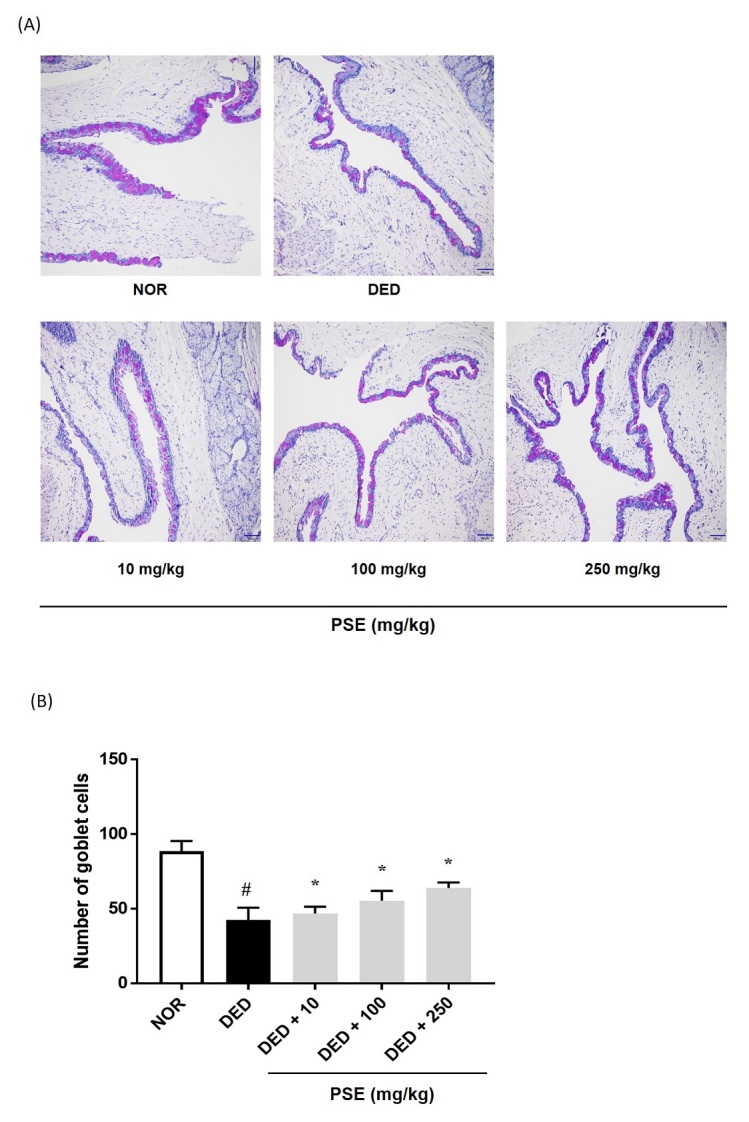
Effects of PSE on conjunctival goblet cells *in vivo*. (A) and (B) Histology by Periodic acid-Schiff (PAS) staining of the conjunctival epithelium in exorbital lacrimal gland-excised rats. The values in the bar graph represent mean ± standard error (SE), n = 5. # statistical difference from the normal group (*p*<0.001), * statistical difference from the vehicle-treated dry eyed group (*p*<0.001).

In the previous studies, DED was demonstrated to induce an inflammatory response, which was caused by the decrease in the number of goblet cells, and increase in inflammatory cytokines^15^. We investigated the effect of PSE on the goblet cell density in the conjunctival tissue of exorbital lacrimal gland-excised rats. As shown in Figure 3A, the goblet cell loss was considerably increased in the DED group, as compared to that in the normal group. However, oral administration of PSE at 100 mg/kg and 250 mg/kg significantly recovered the goblet cells in the DED-induced conjunctival tissue (55.25 ± 6.65 and 63.75 ± 3.77, F=33.76, *p*<0.001) (Figure 3B). Taken together, these data show that PSE has a protective effect in the exorbital lacrimal gland-excised rats through the inhibition of corneal damage and conjunctival goblet cell loss.

**Figure 4.**
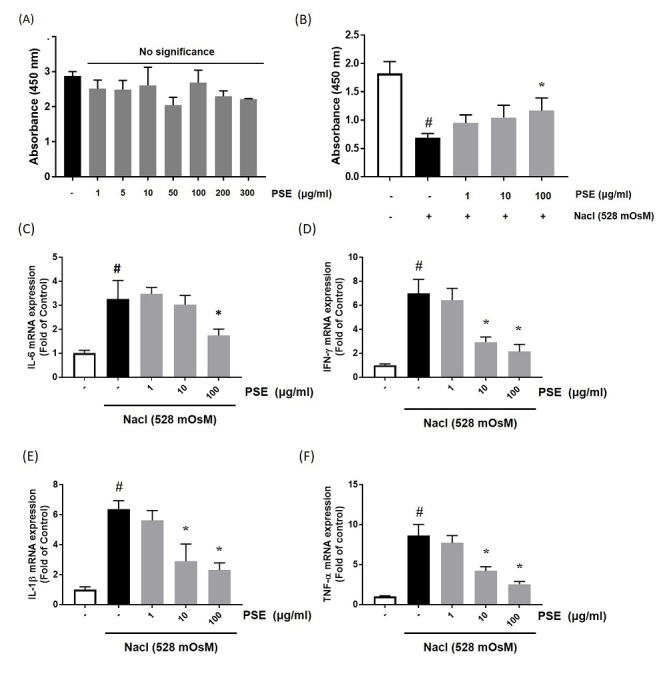
Effects of PSE on hyperosmolar stress-induced inflammation in human conjunctival cells (HCCs). (A) HCCs were treated with PSE (~1-300 μg/ml) for 24 h. (B) HCCs were co treated with PSE (~1-100 μg/ml) and hyperosmotic media for 24 h. To investigate cell viability, CCK-8 assay was performed. The mRNA levels of IL-6 (C), IFN-γ (D), IL-1β (E), and TNF-α (F) were assessed by real-time PCR assay. GAPDH was considered the internal control. Data are the mean ± SEM of three independent experiments for all groups. # statistical difference from the untreated group (*p*<0.001), * statistical difference from hyperosmotic-treated group (*p*<0.001).

To examine the effect of PSE on cytotoxicity in HCCs, we used the CCK-8 kit at the indicated PSE concentration for 24 h. The results indicated that PSE had no cytotoxicity at the indicated concentrations (Figure 4A). To confirm the effect of PSE on DED in vitro, we used hyperosmolar cell culture media with added sodium chloride (528 mOsM) to culture HCCs. The hyperosmolar stress significantly induced cytotoxicity, but the PSE treatment at a concentration of 100 μg/ml markedly restored the cell viability (F=16.04, *p* < 0.001) (Figure 4B). To further investigate the effect of PSE on hyperosmolar-stimulated inflammation, HCCs were co-treated with the hyperosmotic media and the indicated concentrations of PSE. Quantitative real-time PCR demonstrated that the hyperosmolar stress media significantly increased the mRNA expression of Interleukin-6 (IL-6) (F=19.08, *p*<0.001), Interferon- γ (IFN-γ) (F=37.66, *p*<0.0001), Interleukin-1β (IL-1β) (F=33.64, *p*<0.0001) and Tumor necrosis factor-α (TNF-α) (F=49.57, *p*<0.0001). However, PSE treatment considerably inhibited the mRNA expression of each of these genes (Figure 4C-F).

**Figure 5.**
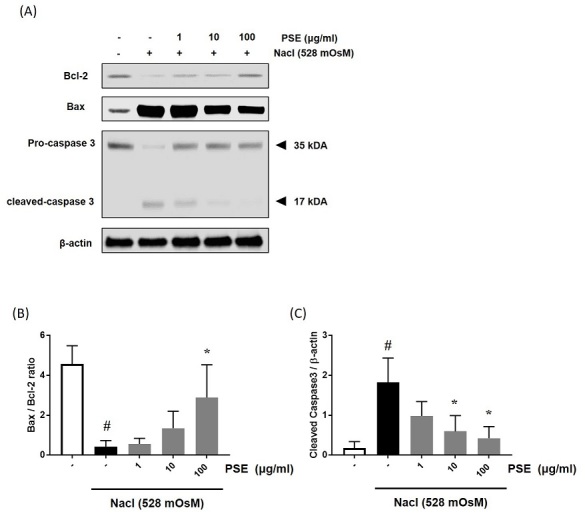
Effects of PSE on hyperosmolar stress-induced apoptotic cell death in human conjunctival cells (HCCs). (A) The protein expression of BAX, Bcl-2, and Caspase 3 was analyzed by western blot analysis. β-actin was used as the loading control. (B), and (C) The relative intensities are expressed as the ratio of BAX, Bcl-2, and cleaved caspase 3 to β-actin. Data are the mean ± SEM of three independent experiments for all groups. # statistical difference from the untreated group (*p*<0.05), * statistical difference from hyperosmotic-treated group (*p*<0.05).

Several studies have demonstrated that hyperosmotic stress initiated the ocular damage activating intrinsic and extrinsic apoptotic pathways^16,17^. Therefore, we examined whether PSE could prevent hyperosmotic stress-induced apoptosis. Hyperosmotic stress triggered apoptotic cell death through increased Bax expression and activation of Caspase-3. However, treatment with PSE significantly inhibited the Bax expression and activation of Caspase-3 (F=8.157, *p*<0.05) and restored Bcl-2 (F=10.4, *p*<0.05) expression in a concentration-dependent manner (Figure 5A-C). These findings show that PSE has a preventive effect via diminishing hyperosmotic stress-induced intrinsic apoptotic pathway in HCCs.

## DISCUSSION

Dry eye is a disorder of the tear film that leads to eye discomfort and irritation, which is caused by the damage to the ocular surface owing to the lack of tears or high evaporation of the tear film^18^. Essentially, a certain amount of tears is constantly produced throughout the day, forming a tear layer in the front of the eyes to gently lubricate the eyes and also to act as a bactericidal agent^19^. However, the lack of these basic tears or fluctuations in their composition causes various unpleasant symptoms, which are referred to as dry eye disease.

The main symptoms of dry eye disease include burning, irritation, pain, foreign body, itching, dryness, glare, bleeding (accumulation of the float), tears, hyperemia, and fatigue. Accompanying eye diseases include conjunctivitis, keratitis, pterygium, ptosis, and may also be accompanied by dry mouth and skin, joint pain, urinary disorders, sexual dysfunction, swallowing disorder, and constipation. In severe cases, the glossy look of the eyeballs disappears and becomes opaque, which can lead to blindness^20^.

There are three major treatments for dry eye disease. The first is the application of artificial tears or ointments to compensate for the lack of tears. Second is the surgical treatment, as performed for fistula occlusion, which is to relieve the symptoms by preventing the tears from running, from the eyes to the nose, for longer management of the lack of tears or hemp eyes. The third is adjuvant therapy, which keeps the surrounding environment at high humidity to prevent the tears from drying out^21^.

We investigated the effect of PSE treatment on exorbital lacrimal gland-excised rats and HCCs. The results show that the inhibition of inflammation by PSE treatment could be beneficial in the treatment of dry eye disease. *In vivo*, PSE restored the tear volume and goblet cell density *via* inhibiting severe corneal irregularities and damage. Treatment with PSE significantly attenuated the hyperosmolar stress-induced inflammation and cell death through suppression of the mRNA expression levels of TNF-α, IL-6, IL-1β, and IFN-γ, and the expression of Bax, as well as activation of caspase-3 *in vitro*.

The inflammatory cytokines on the ocular surface and conjunctival tissues are related to their epithelial disorder; they cause squamous metaplasia of ocular-surface epithelial cells^22^. Especially, IFN-γ attenuates goblet cell differentiation, and TNF-α, IL-6, and IL-1β induce programmed cell death, such as apoptosis and pyroptosis^23,24^. In this study, our results demonstrated that hyperosmotic stress significantly increases the mRNA expression of TNF-α, IL-6, IL-1β, and IFN-γ in HCCs, but treatment with PSE remarkably reduced their mRNA expression in a concentration-dependent manner.

Previous studies indicated that the leaves and roots of P. cuspidatum contain sufficient amino acids, vitamins, and flavonoids that have antibacterial, anti-inflammatory, anti-oxidative, and wound-healing effects^25-27^. The major active components in P. cuspidatum are caftaric acid, polydatin, rutin, quercitrin, and resveratrol. The five active components are well known to be present in many natural plant extracts, and several studies showed their beneficial effects in anti-inflammation and anti-oxidation^28^. In our study, we confirmed that PSE contains a certain amount of polydatin and rutin. It has been demonstrated that hyperosmolar stress could increase intracellular ROS that activates various transcription factors, including the well-known NF-κB, leading to the production of inflammatory cytokines^29^ and NLRP3 inflammasome complex, known as pyroptosis to cause severe dry eye disease^30^. However, polydatin has significant effect in regulating these signaling pathways to treat the dry eye disease. In addition, PSE, including its active components polydatin, is also considered to have positive effects on dry eye disease.

In conclusion, we have indicated that lacrimal gland excision and hyperosmotic stress caused inhibition of tear volume and conjunctival goblet cell damage, and the conjunctival damage is mediated through enhanced inflammation. However, these alterations were recovered by treatment with PSE. Particularly, PSE inhibited hyperosmotic stress-induced cell death in HCCs by inhibiting the mRNA expression of inflammatory cytokines and apoptotic proteins. Therefore, the inhibitory effects of PSE on dry eye disease suggest the possibility of nutritional intervention against inflammatory diseases. In addition, this study proposes that PSE, which has been used as food, is safe to use daily, in a dose-controlled manner without any side effects.
